# Concurrent trajectories of self-rated health and working hour patterns in health care shift workers: A longitudinal analysis with 8-year follow-up

**DOI:** 10.3389/fpubh.2022.926057

**Published:** 2022-09-06

**Authors:** Jenni Ervasti, Laura Peutere, Marianna Virtanen, Oxana Krutova, Aki Koskinen, Mikko Härmä, Mika Kivimäki, Annina Ropponen

**Affiliations:** ^1^Finnish Institute of Occupational Health, Helsinki, Finland; ^2^School of Educational Sciences and Psychology, University of Eastern Finland, Joensuu, Finland; ^3^Division of Insurance Medicine, Karolinska Institutet, Stockholm, Sweden; ^4^Clinicum, University of Helsinki, Helsinki, Finland; ^5^Department of Epidemiology and Public Health, University College London, London, United Kingdom

**Keywords:** night work, health, working hours, multi-trajectory analysis, shift work (MeSH)

## Abstract

**Background:**

The association between health and working hours is hypothesized to be reciprocal, but few longitudinal studies have examined changes in both health and working hour patterns over time. We examined combined trajectories of self-related health and two working hour patterns (working <35 h/week and working night shifts) and the extent to which these trajectories were predicted by employees' lifestyle and mental health.

**Methods:**

Participants of this cohort study with a 8-year follow-up were 5,947 health care shift workers. We linked self-reports of health from three repeated surveys with objective pay-roll based data on working hours. Using group-based multi-trajectory analysis we identified concurrent trajectories for self-rated health and working hour patterns. We examined their associations with baseline lifestyle-related factors (smoking, at-risk alcohol use, obesity, and physical inactivity) and mental health (sleep problems and psychological distress) using multinomial regression analysis.

**Results:**

Three combined trajectories of self-rated health and working <35 h/week and four combined trajectories of self-rated health and night work were identified. Unhealthy lifestyle and poor mental health were associated with trajectories of moderate and declining health. Sleep problems were linked with working <35 h/week. Younger age and good mental health were associated with a combined trajectory of good health and continued night shift work.

**Conclusion:**

Trajectories of suboptimal and declining health are associated with trajectories of reducing working hours and leaving night work, and are more common in employees with unhealthy lifestyle, sleep problems, and psychological distress.

## Introduction

Long working hours (1, 2) and shift work (3–5) may contribute to health problems, but health problems may also affect the choice of working hours and patterns (6, 7). For example, people with health problems are more likely to work part-time (8, 9) and may be less likely to work night shifts, particularly in health care sector (10). Age also affects preference for working long hours and night shifts. Older employees have found to prefer work either shorter shifts or day shifts over longer working hours and nights (11, 12) and it has been suggested that younger and older employees respond differently to long working hours or night work (13, 14), particularly in the long term (15, 16).

However, evidence is limited as most research has relied on study designs where either health or working hour characteristics have been measured on at one point in time only. Due to advances in longitudinal modeling, it would be possible to analyze repeat data on both health and working hour patterns simultaneously. Person-centered approaches, such as growth mixture modeling with longitudinal data, can identify patterns of development and divide participants into qualitatively different latent groups without prior assumptions (17). These latent groups, i.e., developmental trajectories, cannot be directly observed from the data. Providing latent groups, i.e., developmental trajectories, the models are composed of two elements: the probability of group membership and the probability of the observed data given group membership (18). With this method, it is possible to take into account the concurrent development in more than one factor, and approximate the proportion of individuals following specific simultaneous trajectories of working hours and health and examine the antecedents of these trajectories. The trajectories of self-rated health have sparsely been examined before. A Swiss study found slowly declining trajectories not influenced by age, gender, or socioeconomic status (19). A previous study from partly the same cohort as in the current study, achieved somewhat contradicting results showing that a small proportion of employees actually improved their health after transition to retirement. The likelihood of this trajectory was higher in women and in higher socioeconomic status (20). One previous study identified trajectories of working hours and found that female sex, age, lifestyle risk factors, and health problems were associated with short and varying working hours (21). The study did not account for simultaneous changes in self-rated health, and used subjective evaluations on working hours. Thus, little is known about concurrent changes in self-rated health, objectively measured working hours and night work, or the lifestyle determinants of these combined trajectories.

We aimed to examine the concurrent changes in self-rated health and two working hour patterns: working <35 weekly hours and working night shifts. Moreover, we investigated how sex, age, lifestyle factors (smoking, at-risk alcohol use, low physical activity, and obesity), mental health (sleep problems and psychological distress) at baseline were associated with the different identified concurrent trajectories of self-rated health and working hour patterns among shift working health care employees.

## Methods

### Study population

Participants were drawn from the Finnish Public Sector cohort study (22, 23), and they were employees of ten towns, four hospital districts, and two other health care organizations. They were followed up with questionnaire surveys at 2 to 4-year intervals in 2008–2016. Survey responses were linked to records of the participating organizations' registers of payroll-based daily working hour data including information on age and sex, obtained from the shift scheduling program Titania^®^ for each participant. Titania^®^ is an administrative software used at workplaces with shift work. The scheduling program includes both the planned and actual working hour data [for details, see reference (24)]. These were identified based on the starting and ending times of daily working hours using the classification of work shifts in which night shift was determined as ≥ 3 h of work between 23:00 and 06:00 h as previously described (25). Titania^®^ data on annual average weekly working hours and night work on the preceding year to each survey were also linked to survey data. The participants had period-based work contracts organized as day work or shift work, where working hours are planned and balanced for every 3 weeks (total planned working hours 114 h and 45 min). Detailed information is given elsewhere (26), but in general, working hours in the period-based work are mostly irregular. Of the participants, 94% were in shift work and 6% in day work. Day workers may also work occasionally (by demand) other than day shifts.

**Figure 1:**
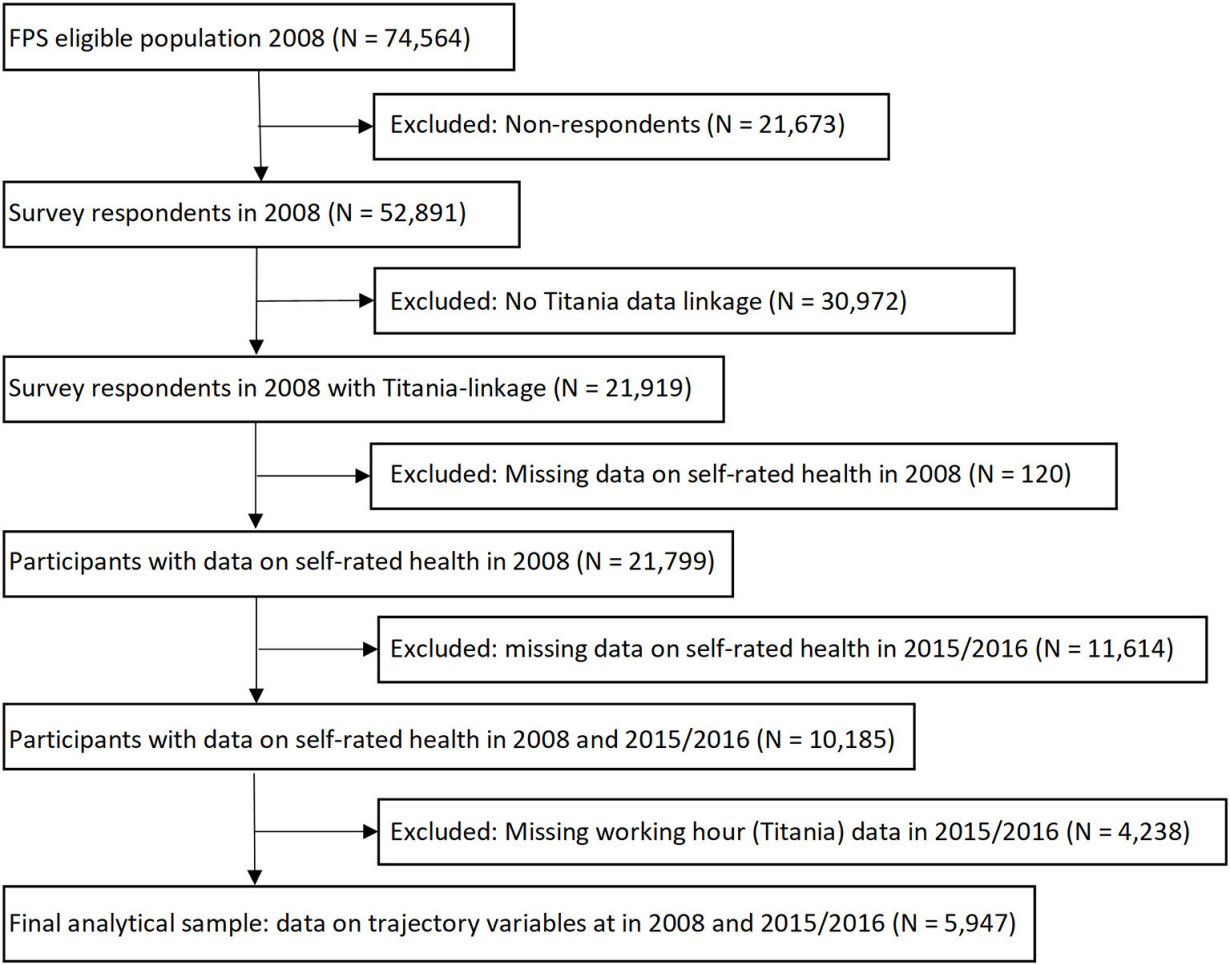
Flow chart of the study participants.

The self-reported data analyzed in the current study included responses to three questionnaire surveys administered in 2008, 2012, and 2015–2016 (average response rate 70%). The baseline was the response given in 2008. Participants with data on self-rated health and working hour patterns for at least in 2008 and 2015–2016 were included in the analysis. This resulted in an analytical sample of 5,947 participants, as described in Figure 1. Ethics approval is from the Ethical Committee of the Helsinki and Uusimaa hospital district (HUS/1210/2016).

### Survey-based measures

To assess self-rated health, we used a single-item measure “How do you rate your health?” with response options; 1 = poor; 2 = fairly poor; 3 = average; 4 = fairly good; 5 = good. The question is widely used and recommended as a standard indicator of health in surveys (27).

Self-reported lifestyle risk factors included smoking, at-risk alcohol use, obesity, and physical inactivity. Smoking was classified into current smoker, never smoker, or ex-smoker (28). Alcohol use was elicited by questions on weekly consumption. One drink was approximately equivalent to one unit or one glass of alcoholic drink or 12 g of alcohol. Alcohol use was dichotomized into no use or moderate use (a maximum of 140 g or 11 units for women and 280 g or 23 units for men) vs. alcohol use greater than this (29). Body mass index (BMI = weight in kilograms divided by height in meters squared) was dichotomized as less than 30 (non-obese) and 30 or more, indicating obesity (30). Participants were categorized as being physically inactive if they reported <2 metabolic equivalent task hours per day (~30 min of walking) and active if more than this (31).

Mental health -related variables were sleep problems, and symptoms of depression and anxiety. We used the Jenkins Sleep Scale to measure common sleep problems during the last month (32, 33). Four items evaluated: the difficulty to fall asleep, wake up at night, difficulty to stay asleep, and wake up exhausted in the morning. Each item is rated on a scale from zero to five, where 0 = never, 1 = 1–3 nights/month, 2 = about 1 night/week, 3 = 2-4 nights/week, 4 = 5-6 nights/week, and 5 = nearly every night. The total score is a sum of all four items' scores from zero (no sleep problems) to 20 (most sleep problems). Participants with a score 4 or higher were coded as having sleep problems (1 = case, 0 = non-case). We used the 12-item General Health Questionnaire (GHQ-12) to measure psychological distress, that is, symptoms of depression and anxiety (34). In GHQ-12, respondents rate the extent to which they are affected by each of the 12 symptoms of distress (0 = not at all, 0 = as much as usual, 1 = slightly more than usual, 1 = much more than usual). Participants with a rating of 1 in at least 4 items of the total measure were coded as cases of psychological distress (1 = case, 0 = non-case).

### Register-based measures

The average weekly (from Monday 00:00 to Sunday 24:00) working hours during the year immediately preceding the survey (= the start date for the year was the date preceding the survey response) were calculated for each survey year. Calendar weeks without work, that is on paid or non-paid leave, were excluded. Those working <35 h per week were coded as cases (=1), and those working more than that as non-cases (=0). The cut-off of 35 h was chosen based on 35 h being the overall mean of the working hours during the years of this study. Only 12% of the study population had a formal part-time job contract. Of them, 23% worked ≥35 h per week, and 77% less than that. Of those with full-time contract, 42% worked ≥35 h per week, and 58% less than that.

In a similar manner, proportion (%) of night shifts from all shifts during the year preceding the survey years were also calculated. Those working >1% of their shifts in night shifts were coded as working night shifts (=1) and those with <1% as working no/minimal number of night shifts (=0). Age was treated as continuous variable and sex was coded as 1 = men, 2 = women.

### Statistical analysis

We used group-based multi-trajectory analysis (18) to identify trajectories of health and working hours separately for (1) self-rated health and working <35 h/week; and (2) self-rated health and night work. Self-rated health was treated as continuous variable and working hour patterns were dichotomous variables. Group-based multi-trajectory modeling is a form of finite mixture modeling to distinguish and describe subpopulations (clusters) existing within the studied population (18). We used a censored (“regular”) normal model of group-based multi-trajectory analysis with linear distribution. The goodness of model fit was judged by running the procedure several times with the number of trajectory clusters starting from one up to five. The Bayesian Information Criterion, Akaike Information Criterion and average posterior probability were used as criteria to confirm the goodness of fit. We used multinomial regression analyses to determine the extent to which baseline health and lifestyle-related factors were associated with the identified clusters.

Multi-trajectory analysis was performed with Stata/IC Statistical Software: Release 17 (StataCorp, College Station, Texas, USA). The additional freely available Stata module ‘traj' was required to conduct group-based trajectory analysis (Jones and Nagin 1999; 2013). SAS software package (version 9.4; SAS Institute, Inc, Cary, North Carolina) was used for regression analyses.

## Results

Of the 74,564 adults in the eligible population, 52,891 (71%) participated in the baseline survey (T1), 30,569 (58%) were successfully linked to Titania register, and 5,947 (19%) had data also 8 years later, at T3, and were included in the analytical sample (Figure 1). The 5,947 employees were predominantly women (93%). Mean age was 43.6 years (SD = 8.6) in 2008. The majority, 56%, were nurses; 26% personal care workers; 9% head nurses/physicians; 6% cleaners and helpers; and 2% were clerical support workers. Participants' occupational position remained largely unchanged during the follow-up.

A total of 81% rated their health as “good” or “rather good” at baseline (mean = 1.78, SD = 0.81). Self-rated health decreased during follow-up, the corresponding figures being 76% (mean = 1.90, SD = 0.86) in 2012, and 73% (mean = 1.99, SD = 0.89) in 2016. A total of 46% worked <35 weekly working hours at baseline. In 2012 and 2016, the corresponding percentages were 40 and 44%, respectively. A total of 38% worked night shifts at baseline. The percentage working night shifts decreased during the follow-up, being 33% in 2012 and 30% in 2016.

**Figure 2:**
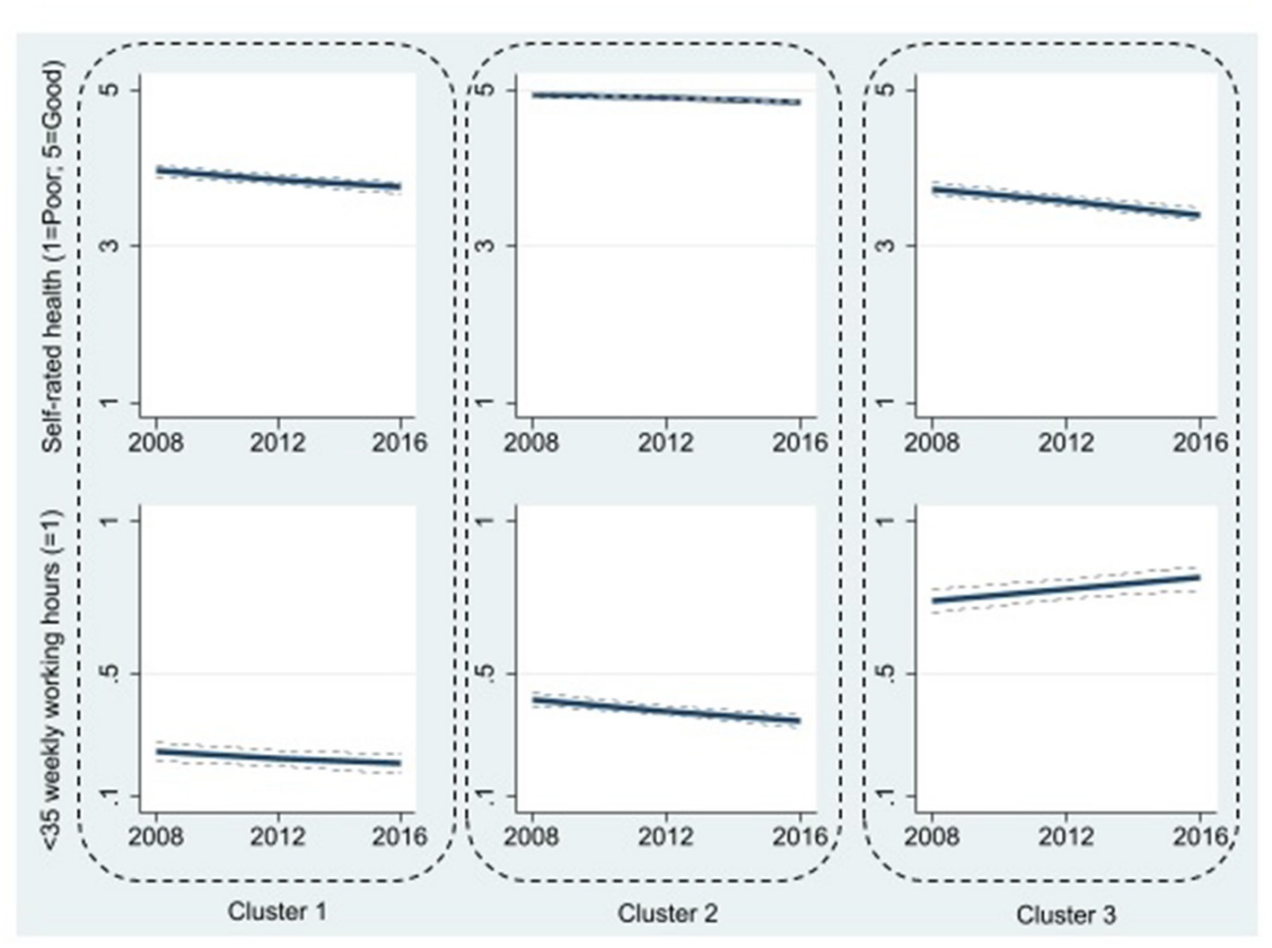
Three clusters of trajectories of self-rated health and working <35 weekly working hours. Cluster 1 (38%): Fairly good but declining self-rated health, and low and decreasing probability of <35 weekly working hours; Cluster 2 (35%): Sustained optimal self-rated health, and moderate but decreasing probability of <35 weekly working hours; Cluster 3 (27%): Fairly good but declining self-rated health, and high and increasing probability of <35 weekly working hours. 95% confidence intervals are shown as dotted lines, albeit they are poorly visible due to being very narrow at times.

A three-cluster model was chosen for working <35 weekly hours, and a four-cluster model for working night shifts (Table 1). The three concurrent trajectories of self-rated health and working <35 h/week were (Figure 2).

1. Cluster 1 (38%): Fairly good but declining self-rated health, and low and decreasing probability of <35 weekly working hours.
2. Cluster 2 (35%): Sustained optimal self-rated health, and moderate but decreasing probability of <35 weekly working hours.
3. Cluster 3 (27%): Fairly good but declining self-rated health, and high and increasing probability of <35 weekly working hours.

**Table 1:** Goodness of fit of group-based trajectory analysis models: the chosen models are shown in bold.

	**Smallest group**				
**Model**	**N**	**%**	**BIC**	**AIC**	**APP**
**Working** ** <35 h/week**					
1-cluster model	5,947	100	−34,880.6	−34,863.9	
2-cluster model	2,158	36.3	−32,534.3	−32,500.9	0.93/0.94
3-cluster model	**1,617**	**27.3**	**−32,074.2**	**−32,024**	**0.91/0.84/0.82**
4-cluster model	Did not converge				
**Working night shifts**					
1-cluster model	5,947	100	−34,094.2	−34,077.5	
2-cluster model	2,004	33.7	−30,311.2	−30,277.8	0.99/0.99
3-cluster model	1,332	22.4	−28,955.1	−28,904.9	0.91/0.93/0.96
4-cluster model	**774**	**13.0**	**−28,062.8**	**−27,995.9**	**0.93/0.92/0.90/0.94**
5-cluster model	Did not converge				

BIC, Bayesian Information Criterion; AIC, Akaike Information Criterion; APP, Average Posterior Probability.

Table 2 shows the associations of lifestyle and mental health -related factors and cluster membership (self-rated health and working <35 weekly hours) based on multinomial regression analysis. Cluster 1 “Fairly good but declining self-rated health, and low and decreasing probability of <35 weekly working hours” was used as a reference cluster.

**Table 2:** Lifestyle and mental health -related factors associated with cluster membership (self-rated health and working <35 weekly hours).

	**Cluster 2: Sustained optimal self-rated**		**Cluster 3: Fairly good but declining self-rated**	
	**health, and moderate but decreasing probability**		**health, and high and increasing probability**	
	**of**<**35 weekly working hours**, ***n*** = **2,083**		**of**<**35 weekly working hours**, ***n*** = **1,555**	
	**OR**	**95% CI**	**OR**	**95% CI**
Men	1		1	
Women	1.23	0.97–1.56	**1.69**	**1.28–2.22**
Age/1 year	**0.93**	**0.92–0.94**	**0.96**	**0.95–0.97**
Non-smoking	1		1	
Ex-smoking	**0.77**	**0.65–0.92**	0.98	0.82–1.17
Smoking	**0.61**	**0.50–0.74**	1.06	0.88–1.27
No at-risk alcohol use	1		1	
At-risk alcohol use	0.87	0.68–1.12	0.91	0.72–1.16
Non-obese	1		1	
Obese	**0.41**	**0.33–0.50**	1.12	0.95–1.33
≥ 2 MET hr/day	1		1	
<2 MET hr /day	**0.59**	**0.50–0.70**	1.03	0.88–1.20
No sleep problems	1		1	
Sleep problems	**0.51**	**0.43–0.61**	**1.17**	**1.00–1.37**
No psychological distress	1		1	
Psychological distress	**0.46**	**0.39–0.55**	1.16	0.99–1.35

Multinomial regression (reference=Cluster 1 ‘Fairly good but declining self-rated health, and low and decreasing probability of <35 weekly working hours', n = 2,124).

Odds ratio (OR) with 95% confidence intervals (CI). Statistically significant (*p* <0.05) estimates are bolded.

Cluster 2 was characterized by better self–rated health but higher probability of <35 weekly working hours than in the reference cluster, was more probable with less lifestyle risk factors (OR_currentsmoking_ = 0.61, 95% CI 0.50–0.74; OR_ex−smoking_ = 0.77, 95% CI 0.65–0.92; OR_obesity_ = 0.41, 95% CI 0.33–0.50; OR_lowphysicalactivity_ = 0.59, 95% CI 0.50–0.70), less sleep problems (OR = 0.51, 95% CI 0.43–0.61), and less psychological distress (OR = 0.46, 95% CI 0.39–0.55). Employees in Cluster 2 were younger than those in Cluster 1 (OR = 0.93, 95% CI 0.92–0.94).

Compared to Cluster 1, Cluster 3 was characterized by similar fairly good but declining self–rated health, but a higher and increasing probability of <35 weekly working hours. Cluster membership in Cluster 3 was more probable in women (OR = 1.69, 95% CI 1.28–2.22) and younger employees (OR = 0.96, 95% CI 0.95–0.97). Clusters 1 and 3 did not differ by lifestyle factors, but employees in Cluster 3 had a higher likelihood of sleep problems (OR = 1.17, 95% CI 1.00–1.37) than those in Cluster 1.

**Figure 3:**
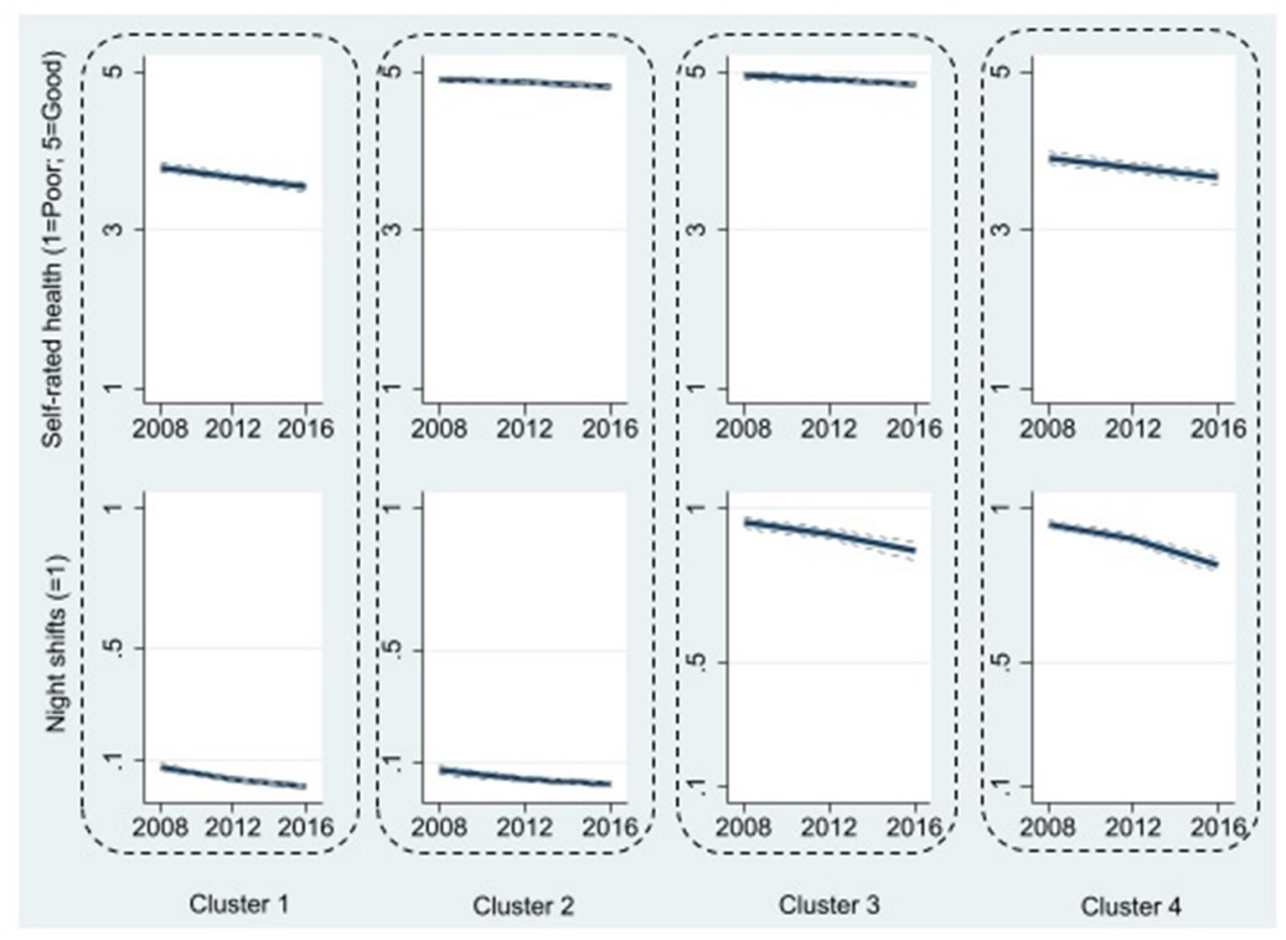
Four clusters of trajectories of self-rated health and working night shifts. Cluster 1 (41%): Fairly good but declining self-rated health, and no night work; Cluster 2 (25%): Sustained optimal self-rated health, and no night work; Cluster 3 (13%): Sustained optimal self-rated health, and high but slightly decreasing probability of night work; Cluster 4 (21%): Fairly good but declining self-rated health, and high but decreasing probability of night work. 95% confidence intervals are shown as dotted lines, albeit they are poorly visible due to being very narrow.

The four concurrent trajectories of self-rated health and working night shifts were (Figure 3).

1. Cluster 1 (41%): Fairly good but declining self-rated health, and no night work.
2. Cluster 2 (25%): Sustained optimal self-rated health, and no night work.
3. Cluster 3 (13%): Sustained optimal self-rated health, and high but slightly decreasing probability of night work.
4. Cluster 4 (21%): Fairly good but declining self-rated health, and high but decreasing probability of night work.

Table 3 shows the associations of lifestyle and mental health -related factors and cluster membership (self-rated health and night shifts) based on multinomial regression analysis. Cluster 2 “Sustained optimal self-rated health, no night work” was used as a reference cluster.

**Table 3:** Health and lifestyle -related factors associated with cluster membership (self-rated health and night work).

	**Cluster 1: Fairly good but**		**Cluster 3: Sustained optimal**		**Cluster 4: Fairly good but**	
	**declining self-rated health, and**		**self-rated health, and high**		**declining self-rated health**,	
	**no night work**, ***n*** = **2,443**		**but slightly decreasing**		**and high but decreasing**	
			**probability of night**		**probability of night**	
			**work**, ***n*** = **755**		**work**, ***n*** = **1,183**	
	**OR**	**95% CI**	**OR**	**95% CI**	**OR**	**95% CI**
Men (=ref)	1		1		1	
Women	1.03	0.78–1.36	0.90	0.65–1.26	0.81	0.60–1.10
Age/1 year	**1.05**	**1.04–1.06**	**0.95**	**0.94–0.96**	1.00	0.99–1.01
Non-smoking	1		1		1	
Ex-smoking	**1.38**	**1.14–1.67**	**1.31**	**1.02–1.69**	**1.36**	**1.09–1.70**
Smoking	**1.96**	**1.55–2.48**	**2.16**	**1.62–2.67**	**3.13**	**2.45–4.01**
No at-risk alcohol use	1		1		1	
At-risk alcohol use	0.96	0.75–1.24	**0.47**	**0.31–0.73**	0.91	0.67–1.22
Non-obese	1		1		1	
Obese	**2.81**	**2.22–3.57**	1.29	0.92–1.80	**3.23**	**2.49–4.17**
≥ 2 MET hr/day	1		1		1	
<2 MET hr /day	**1.74**	**1.46–2.08**	0.96	0.75–1.23	**1.45**	**1.19–1.78**
No sleep problems	1		1		1	
Sleep problems	**2.17**	**1.79–2.63**	1.07	0.81–1.42	**1.93**	**1.55–2.41**
No psychological distress	1		1		1	
Psychological distress	**2.24**	**1.86–2.70**	**0.74**	**0.56–0.99**	**1.99**	**1.61–2.45**

Multinomial regression (ref=Cluster 2 ‘Sustained optimal self-rated health, no night work', n = 1,381).

Odds ratio (OR) with 95% confidence intervals (CI). Statistically significant (*p* <0.05) estimates are bolded.

Cluster 1, characterized by poorer and declining self-rated health than in the reference cluster, but similar minimal levels of night work' was more probable among older employees (OR = 1.05, 95% CI 1.04–1.06), in those with more lifestyle risk factors (OR_currentsmoking_ = 1.96, 95% CI 1.55–2.48; OR_ex−smoking_ = 1.38, 95% CI 1.14–1.67; OR_obesity_ = 2.81, 95% CI 2.22–3.57; OR_lowphysicalactivity_ = 1.74, 95% CI 1.46–2.08), sleep problems (OR = 2.17, 95% CI 1.79–2.63), and psychological distress (OR = 2.24, 95% CI 1.86–2.70).

Cluster 3, characterized by similar optimal self–rated health, but contrary to the reference cluster, employees worked night shifts, was more probably among younger employees (OR = 0.95, 95% CI 0.94–0.96) and those who smoked (OR_current_ = 2.16, 95% CI 1.62–2.87; OR_ex−smokers_ = 1.31, 95% CI 1.02–1.69), but also those with a lower likelihood of at–risk alcohol use (OR = 0.47, 95% CI 0.31–0.73) and psychological distress (OR = 0.74, 95% CI 0.56–0.99).

Cluster 4, characterized by both poorer self–rated health and higher probability of night work, was associated with a higher likelihood of lifestyle risk factors (OR_currentsmoking_ = 3.13, 95% CI 2.45–4.01; OR_ex−smoking_ = 1.36, 95% CI 1.09–1.70; OR_obesity_ = 3.23, 95% CI 2.49–4.17; OR_lowphysicalactivity_ = 1.45, 95% CI 1.19–1.78), sleep problems (OR = 1.93, 95% CI 1.55–2.41), and psychological distress (OR = 1.99, 95% CI 1.61–2.45).

## Discussion

This study examined the combined trajectories of self-rated health and objectively measured working hour patterns over an 8-year follow-up among 5,947 shift working healthcare employees. Moreover, we examined lifestyle and mental health -related factors that predicted each trajectory. For self-rated health and working <35 hours/week, three distinctive clusters emerged: (1) “Slightly declining good health, and slightly increasing working time”; (2) “Sustained good health, and moderate but slightly increasing working time”; and (3) “Fairly good but declining health, short and decreasing working hours”.

The identified concurrent trajectories of health and patterns of working hours add to the earlier research on healthy worker effect (35). Our results corroborate the previous findings that working hour patterns are modified by health (1–7). A total of 27% of participants belonged to the cluster characterized by declining health and shortening weekly working hours. This is in line with earlier findings that declining health is often associated with working fewer hours (8, 9). However, this is not the case for all employees: 38% worked standard hours despite declining health. Congruent to earlier studies, lifestyle risk factors were predictive of poorer self-rated health (36). However, sleep problems were the only risk factor linked with high and stable probability short weekly working hours. Previous research has shown significant differences between individuals in sleepiness (37), which may affect their suitability to shift work.

For self-rated health and night shift work, four distinctive clusters emerged: (1) “Fairly good but declining health, no night work”; (2) “Sustained good health, no night work”; (3) “Sustained good health with night work”; and (4) “Fairly good but declining health with night work”. In both trajectories including night work, the trend was decreasing. We did not identify trajectories, or trajectory clusters, characterized by increasing trend in night work. A total of 66% of participants belonged to the two clusters characterized by good health, but a minimal amount night work. Also here, lifestyle risk factors were predictive of poorer health. Those who continued working night shifts despite declining health, had a higher probability of poor lifestyle and mental health as compared to those also having declining health, but not working night shifts. In turn, those who remained in optimal health and consistently worked night shifts for the entire follow-up of 8 years, were characterized by younger age, lower likelihood of at-risk alcohol use, and lower likelihood of psychological distress.

The association with younger age was expected as earlier studies have shown that younger and older employees might respond differently to night work (13, 14). However, few longitudinal studies on the association between night shift work and lifestyle factors exist. Night shift work has been previously associated with poor sleep patterns, higher body mass index and smoking (38–41). The results on physical activity have been mixed (40, 42), but a previous study originating from the same Finnish public sector cohort as used in this study, showed an increased probability of physical activity in employees with night shifts (43).

Our findings support the hypothesis of the healthy worker effect in night work (35), that is, those who have health problems might be less likely to work night shifts or move from night work to day work (10). We identified a distinct cluster characterized by employees with suboptimal health selected to daywork, and another district cluster characterized by employees with sustained optimal health selected to night work. Between these clusters, there were employees characterized by decreasing trend in both probability of night shift work and in health. This cluster can be described as comprising those among whom the adverse effects of continuing night work are best illustrated. Our findings support earlier research findings showing that older employees prefer shorter shifts and day shifts (11), and that the benefits to improved sleep quality are most pronounced among aging employees transferring from shift work to daywork (25).

Depending on the multi-trajectory model, 35–38% of participants had a sustained optimal health status. For the majority (62–65%), the health trajectory was declining. However, as the sample consisted of employed working-aged individuals, self-rated health status was not poor in any of the clusters. There were, however, no clusters where health would improve in line with less working hours or less night shifts. Some earlier studies have examined the trajectories of self-rated health in varying populations, and in found similarly shaped trajectories (44, 45).

The study is the first to examine the concurrent changes in self-rated health and objectively measured working hour patterns. The use of objective data on working hours is a strength and adds validity to our findings, as subjective estimates on working hours are prone to recall or reporting bias. Our findings may be of specific interest for the health care sector struggling with aging employees, personnel turnover and shortage, and both physically and psychosocially demanding work (46, 47), as they support other studies on shift work and health proposing that employees over 50 years should be offered an opportunity to move away from shift work to avoid health problems (48, 49).

Two main limitations of this study are data attrition (linkage to working hour records was available for 21,919 out of 52,891 respondents but due to missing data at follow-up the final analytic sample included only 5,947 participants) and generalizability given that 93% were women, 56% nurses and all were working in the healthcare sector. The loss to follow-up, particularly in survey responses in 8 years from 2008 to 2015/2016, may partly be due to employees changing employers, or having left the labor market either temporarily or permanently. The possible health-related selection out of the labor market suggests that our estimates may be underestimates of the true effect. That is, without any health-related selection to another employer or out of the labor market, the differences in trajectories would be even more pronounced, and subsequent associations with lifestyle and mental health stronger. Moreover, we only had an opportunity to measure self-rated health and working hour patterns in three timepoints. However, the follow-up time on 8 years was rather long. The cohort included predominantly women working in public health care sector in Finland. Therefore, the results may not be generalizable to male-dominated private sector employees in shift work. Finally, as the trajectories are approximations of the true development, we cannot rule out that some people were misclassified and the group where they were placed does not describe the true development of their health and working time patterns. However, as the reliabilities in each trajectory group were satisfactory (average posterior probability ranged from 0.82 to 0.94), a classification error is an unlikely source of major bias in our results.

To conclude, the results of this study indicate that suboptimal and declining health is linked with sub-standard working hours and potential transition away from night shifts, which may indicate the healthy worker effect. These results may partly explain, why some studies have struggled to find associations between working hour patterns and sickness absence.

## Data availability statement

The datasets presented in this article are not readily available because linked working hour data records require separate permission from the data owners. Statistical syntax for the analysis of the present study is available from the corresponding author. Anonymized questionnaire data from the FPS study can be shared upon request to the corresponding author. Requests to access the datasets should be directed to jenni.ervasti@ttl.fi.

## Ethics statement

The studies involving human participants were reviewed and approved by Ethical Committee of the Helsinki and Uusimaa Hospital district (HUS/1210/2016). Written informed consent for participation was not required for this study in accordance with the national legislation and the institutional requirements.

## Author contributions

JE, LP, and AR designed the study. AK managed the data. JE performed data analysis and wrote the first draft. All authors participated in interpreting the data and critically reviewing the paper.

## Funding

The Academy of Finland, DIGIHUM-programme grants (329200, 329201, and 329202).

## Conflict of interest

The authors declare that the research was conducted in the absence of any commercial or financial relationships that could be construed as a potential conflict of interest.

## Publisher's note

All claims expressed in this article are solely those of the authors and do not necessarily represent those of their affiliated organizations, or those of the publisher, the editors and the reviewers. Any product that may be evaluated in this article, or claim that may be made by its manufacturer, is not guaranteed or endorsed by the publisher.
